# The effects of antimicrobial peptides buCaTHL4B and Im-4 on infectious root canal biofilms

**DOI:** 10.3389/fbioe.2024.1409487

**Published:** 2024-08-16

**Authors:** Ziqiu Hu, Haixia Ren, Yifan Min, Yixin Li, Yuyuan Zhang, Min Mao, Weidong Leng, Lingyun Xia

**Affiliations:** ^1^ Department of Stomatology, Taihe Hospital, Hubei University of Medicine, Shiyan, China; ^2^ Institute of Oral Diseases, School of Dentistry, Hubei University of Medicine, Shiyan, China; ^3^ Department of Stomatology, Zhushan County People’s Hospital, Shiyan, China

**Keywords:** antimicrobial peptide, buCaTHL4B, Im-4, apical periodontitis, *Enterococcus faecalis*, *Fusobacterium nucleatum*

## Abstract

**Purpose:**

The primary cause of pulp and periapical diseases is the invasion of bacteria into the root canal, which results from the continuous destruction of dental hard tissues. Effective management of infections during root canal therapy necessitates effectively irrigation. This study aims to investigate the effects of two antimicrobial peptides (AMPs), buCaTHL4B and Im-4, on root canal biofilms *in vitro*.

**Methods:**

Two-species biofilms (*Enterococcus faecalis* and *Fusobacterium nucleatum*) were selected and anaerobically cultivated. The following treatments were applied: 10 μg/mL buCaTHL4B, 10 μg/mL Im-4, 5 μg/mL buCaTHL4B, 5 μg/mL Im-4, 1 μg/mL buCaTHL4B, 1 μg/mL Im-4, 1% NaOCl, and sterile water. Each group was treated for 3 min. Subsequently, the two strains were co-cultured with 10 μg/mL buCaTHL4B, 10 μg/mL Im-4, 1% NaOCl, and sterile water for 24, 48, and 72 h. The biofilms were examined using confocal laser scanning microscopy (CLSM) with fluorescent staining, and the percentages of dead bacteria were calculated. Quantitative real-time PCR (qRT-PCR) was employed to assess the variations in bacterial proportions during biofilm formation.

**Results:**

Compared to 1% NaOCl, 10 μg/mL buCaTHL4B or Im-4 exhibited significantly greater bactericidal effects on the two-species biofilms (*p* < 0.05), leading to their selection for subsequent experiments. Over a 48-hour period, 10 μg/mL Im-4 demonstrated a stronger antibiofilm effect than buCaTHL4B (*p* < 0.05). Following a 24-hour biofilm formation period, the proportion of *F. nucleatum* decreased while the proportion of *E. faecalis* increased in the sterile water group. In the buCaTHL4B and 1% NaOCl groups, the proportion of *F. nucleatum* was lower than that of *E. faecalis* (*p* < 0.05), whereas in the Im-4 group, the proportion of *F. nucleatum* was higher than that of *E. faecalis* (*p* < 0.05). The proportions of bacteria in the two AMPs groups gradually stabilized after 24 h of treatment.

**Conclusion:**

buCaTHL4B and Im-4 exhibited remarkable antibacterial and anti-biofilm capabilities against pathogenic root canal biofilms *in vitro*, indicating their potential as promising additives to optimize the effectiveness of root canal treatment as alternative irrigants.

## 1 Introduction

Intact dental hard tissues effectively protect the internal pulp tissues. However, when these hard tissues are destroyed, the invasion of microorganisms can lead to pulp necrosis, followed by periapical lesions (An et al., 2012). Periapical periodontitis is a challenging oral infectious disease, often associated with *E. faecalis* and *F. nucleatum*. *Enterococcus faecalis* is a facultative anaerobic bacterium capable of long-term survival in the root canal due to its resistance to host immunity and various antibacterial treatments (Zhu et al., 2010; Lins et al., 2013; Barbosa-Ribeiro et al., 2016; Bouillaguet et al., 2018). Numerous studies have shown that *F*. *nucleatum* is frequently found in high prevalence and abundance, primarily linked to primary endodontic infections (Mussano et al., 2018; Hu Z. et al., 2023). As a “bridge bacterium”, *F*. *nucleatum* co-aggregates with most oral bacteria via various cell surface adhesins, contributing to biofilm formation (Haney et al., 2019a; Manoil et al., 2020; Gomes et al., 2021). Additionally, it is reported that *E. faecalis* and *F. nucleatum* can co-adhere, supporting bacterial survival in unfavorable environments, encouraging interspecies communication, and facilitating biofilm production (Yap et al., 2014). These findings prompted us to create a biofilm model resembling those in the infected root canal, using *E. faecalis* and *F. nucleatum* as the dominant species. This biofilms formation is particularly relevant in dental research, they provide insights into how biofilms respond to various disinfection strategies, ensuring that the findings are applicable to actual dental practice.

Research into efficient root canal irrigants that suppress bacterial biofilm is essential to reduce periapical irritation and increase treatment success rates. During the preparation of an infected root canal, sodium hypochlorite (NaOCl), a traditional irrigating solution, is beneficial due to its ability to destroy necrotic tissue and its broad-spectrum antibacterial qualities. (Clarkson and Moule, 1998; Barakat et al., 2024). However, improper use of NaOCl can alter dentinal microhardness and bond strength, and degrade the collagen structure of dentin (Slutzky-Goldberg et al., 2004). Extrusion of NaOCl into periapical tissues may result in pain (Huang et al., 2019). Therefore, there is an urgent need to develop alternative irrigant additives that can effectively inhibit biofilms.

Antimicrobial peptides (AMPs) are effector molecules of innate defense systems. These small molecule products, typically composed of 12–60 amino acids, 2 to 9 positive charges, and an amphiphilic structure, are produced by single genes (Abdi et al., 2019; Grimsey et al., 2020). Antimicrobial peptides may attach to bacterial membranes via cations, causing damage to the membrane through the formation of barrel-stave, carpet, and toroidal pore model structures (Khurshid et al., 2016). By permeabilizing the cell membrane and preventing DNA or protein production, AMPs inhibit bacterial activity (Raheem and Straus, 2019). Additionally, AMPs can target and prevent bacterial biofilm formation (Wang et al., 2018). It has been discovered that peptide 1018 and DJK-5 inhibit a crucial signal molecule (P)ppGpp, involved in biofilm production (de la Fuente-Nunez et al., 2014; Hu J. et al., 2023). To address the limitations of traditional irrigants, AMPs may be employed as agents for suppressing root canal biofilms (Haney et al., 2019b).

In our previous investigation, buCaTHL4B and Im-4 were identified as efficient antibacterial peptides against dental plaque biofilms. buCaTHL4B exhibits significant bactericidal effects with minimal cytotoxicity, distinguished by its high tryptophan concentration. It causes bacterial membranes to rupture rapidly, resulting in noticeable changes such as foaming, budding, and the creation of pore-like structures (Brahma et al., 2015). Im-4, an immune peptide produced by *Drosophila* upon activation of the Toll innate immune system during defense against fungal infections, was found to be particularly effective in reducing biofilm formation. Im-4 shows increased inhibitory effects on filamentous fungi compared to yeasts, Gram-positive bacteria, and Gram-negative bacteria (Cohen et al., 2020). However, uncertainty persists regarding the specific characteristics and effects of these two AMPs on root canal biofilms.

The purpose of this study was to create a type of two-species biofilms with *E. faecalis* and *F. nucleatum*. The antibacterial properties of buCaTHL4B and Im-4 at different concentrations were examined *in vitro* on these formed biofilms. Analyses were also conducted on the impact of two AMPs on biofilm production and the proportion of bacteria during the biofilm development process. The null hypothesis was that: there is no significant difference in the antimicrobial efficacy between buCaTHL4B, Im-4 at different concentrations and NaOCl against the two-species biofilms.

## 2 Materials and methods

### 2.1 Antimicrobial peptides synthesis

Peptide buCaTHL4B (AIPWIWIWRLLRKG) and Im-4 (FIGMIPGLIGGLISAIK-NH_2_) were synthesized by Sangon Biotech (Shanghai, China) using solid-phase 9-fluorenyl methoxycarbonyl (Fmoc) and purified to 98% using reverse-phase high-performance liquid chromatography (HPLC). The structures and sequences of buCaTHL4B and Im-4 were shown in Supplementary Figure S1. The peptides were resuspended in deionized water and utilized in the present experiments. All stocks remained sterile throughout the duration of the study.

### 2.2 Culture and growth detection of bacteria

*Enterococcus faecalis* (ATCC29212) and *F. nucleatum* (ATCC10953) were employed in this study. Bacterial culture conditions were adapted from a previous study (Huang et al., 2015). The strains were subcultured on Brain Heart Infusion (BHI; BectonDickinson, Sparks, MD) agar plates supplemented with 0.5% yeast extract (YE; OXOID, Hampshire, United Kingdom) and 5% defibrillated sheep blood (Solarbio, Beijing, China). The planktonic strains were proliferated in BHI liquid medium containing 0.5% YE. Both bacterial species were incubated at 37°C under anaerobic conditions.

The bacterial suspension of *E. faecalis*, *F. nucleatum*, and the mixed bacteria in equal volumes were adjusted to an optical density at 600 nm (OD _600_) of 0.10. This was determined using a microplate reader (SpectraMaxi3x, Molecular Devices, United States) in a 96-well plate. Subsequently, the bacterial solutions were diluted tenfold and 150 μL of each bacterial suspension was dispensed into each well of the 96-well plate, with three replicates per bacterial suspension. The plate was then incubated under anaerobic conditions at 37°C, and the OD _600_ was measured every 2 h.

### 2.3 Minimal inhibitory concentration

The minimum inhibitory concentrations (MIC) of buCaTHL4B and Im-4 were determined using the broth microdilution method. The MIC was defined as the peptide concentration at which no bacterial growth was observed. The bacterial suspension of *E. faecalis*, *F. nucleatum* were adjusted to a final concentration of 5 × 10^5^ CFU/mL and added to a 96-well plate, with 100 μL per well. Peptides buCaTHL4B or Im-4 were added to sterile 96-well polypropylene microtiter plates at increasing concentrations (0, 10, 20, 40, and 80 μg/mL), with each concentration tested in triplicate, 10 μL per well. The plates were incubated at 37°C for 24 h, and the absorbance at 630 nm was measured using a microplate reader after 24-hour treatment. Three repeated tests were accomplished for the MIC test.

### 2.4 Biofilm model

Sterile hydroxyapatite (HA) disks (12 mm in diameter and 2 mm in thickness; Bayamon Bioactive Materials Ltd., Chengdu, China) were used as substrates for biofilm growth. The HA disks were coated with 1 mL of type I collagen solution (10 mg/mL collagen in 0.012M HCl in double-distilled water; Biosharp, Hefei, China) in 24-well plates and incubated overnight at 4°C. The bacterial suspension of *F. nucleatum* and *E. faecalis* was mixed in equal volumes, adjusted to an OD _600_ of 0.10, and then diluted tenfold for biofilm culture. The bacterial biofilms were grown in BHI liquid medium containing 0.5% YE and 1% sucrose (Solarbio, Beijing, China).

### 2.5 Antimicrobial peptides treat on preformed biofilms

**FIGURE 1:**
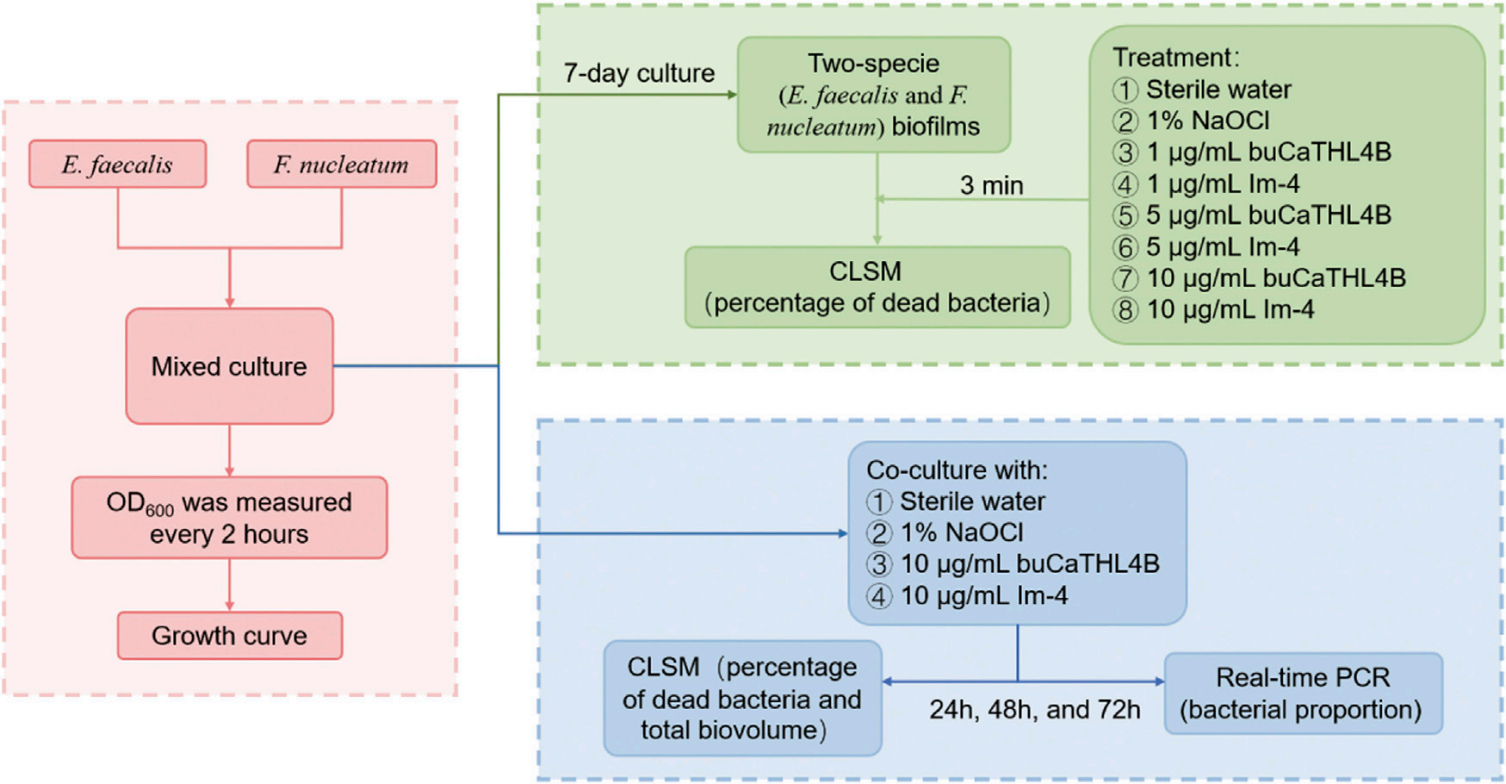
The workflow diagram of this study.

Figure 1 presents the workflow diagram of this study. The two-species biofilms were incubated anaerobically at 37°C on the pre-treated HA disks for 7 days. The disks were then divided into eight treatment groups: (a) sterile water, (b) 1% NaOCl, (c) 1 μg/mL buCaTHL4B, (d) 1 μg/mL Im-4, (e) 5 μg/mL buCaTHL4B, (f) 5 μg/mL Im-4, (g) 10 μg/mL buCaTHL4B, and (h) 10 μg/mL Im-4. Each group contained three disks and treated for 3 min. The test was repeated three times.

### 2.6 Biofilm inhibition test

The disks were divided into four treatment groups: i) sterile water, ii) 1% NaOCl, iii) 10 μg/mL buCaTHL4B, and iv) 10 μg/mL Im-4. Each group contained three disks. The treatments were added to the mixed bacterial suspensions at the onset of biofilm development and maintained for 3 days under anaerobic incubation at 37°C. The final concentration of the AMPs in the bacterial suspension was 10 μg/mL. The blank group received equal amounts of sterile water, while the positive control group received equal amounts of 1% NaOCl. The disks were subjected to the respective treatments at 24, 48, and 72 h. The test was repeated three times.

### 2.7 Confocal laser scanning microscopy examination of biofilms

The biofilms on the HA disks were stained using the LIVE/DEAD BacLight Bacterial Viability Kit L-7012 (Molecular Probes, Eugene, OR, United States) for microscopy and quantitative assays following exposure to the different treatments mentioned above (Huang et al., 2019; Yu et al., 2022). Bacteria with intact cell membranes were stained green by SYTO 9, while bacteria with damaged cell membranes were stained red by propidium iodide (PI). Images of the stained samples were captured using confocal laser scanning microscopy (CLSM; FV3000RS, OLYMPUS, Japan). The excitation wavelengths for SYTO 9 and PI were 488 nm and 561 nm, respectively. Four random areas of the biofilm on each disk were scanned, with 50–70 slices of 2.0 μm collected in each area from the top to the bottom of the biofilm. Imaris 9.0.1 software (Bitplane, Zurich, Switzerland) was used for three-dimensional reconstruction and quantitative analysis of each image. The volume ratio of red fluorescence to the total fluorescence (green and red) indicated the percentage of dead bacteria.

### 2.8 Quantitative real-time PCR

Biofilms co-cultured with AMPs for 24, 48, and 72 h were collected and re-suspended in BHI. The genomic DNA of bacteria was extracted using the Solarbio Bacterial Genomic DNA Extraction Kit (Solarbio, Beijing, China). DNA concentrations were measured with a micro-ultraviolet spectrophotometer (Nanodrop 2000; Thermo, United States). Relevant literature was consulted to determine primers, and the BLAST tool on the NCBI website (http://blast.ncbi.nlm.nih.gov/Blast.cgi) was used to confirm primer specificity for each strain. The primers were as follows: *F. nucleatum*: forward primer GGA​TTT​ATC​TTT​GCT​AAT​TGG​GGA​AAT​TAT​AG, reverse primer ACT​ATT​CCA​TAT​TCT​CCA​TAA​TAT​TTC​CCA​TTA​GA. *Enterococcus faecalis*: forward primer ACC​CCG​TAT​CAT​TGG​TTT, reverse primer ACG​CAT​TGC​TTT​TCC​ATC. A total of 100 ng DNA from each strain was amplified using species-specific primers (0.4 μM) and DNA Taq Polymerase (TAKARA, TB Green Premix Ex Taq II, Japan). PCR protocol included an initial step at 94°C 5 min, followed by 30 cycles of amplification (94°C for 30 sec, 55°C for 30 sec, and 72°C for 30 sec), and a final elongation step at 72°C for 10 min. Bacteria proportions in the biofilms were calculated using the bacterial quantification algorithm proposed by Livak and Schmittgen, (2001); Huang et al. (2015).

### 2.9 Statistical analysis

Statistical analysis was conducted using SPSS Statistics 26.0 (IBM Corp, NY, United States). One-way analysis of variance (ANOVA) with *post hoc* pairwise comparisons was performed, and statistical significance was set at *p* < 0.05.

## 3 Results

### 3.1 Bacterial growth curve

**FIGURE 2:**
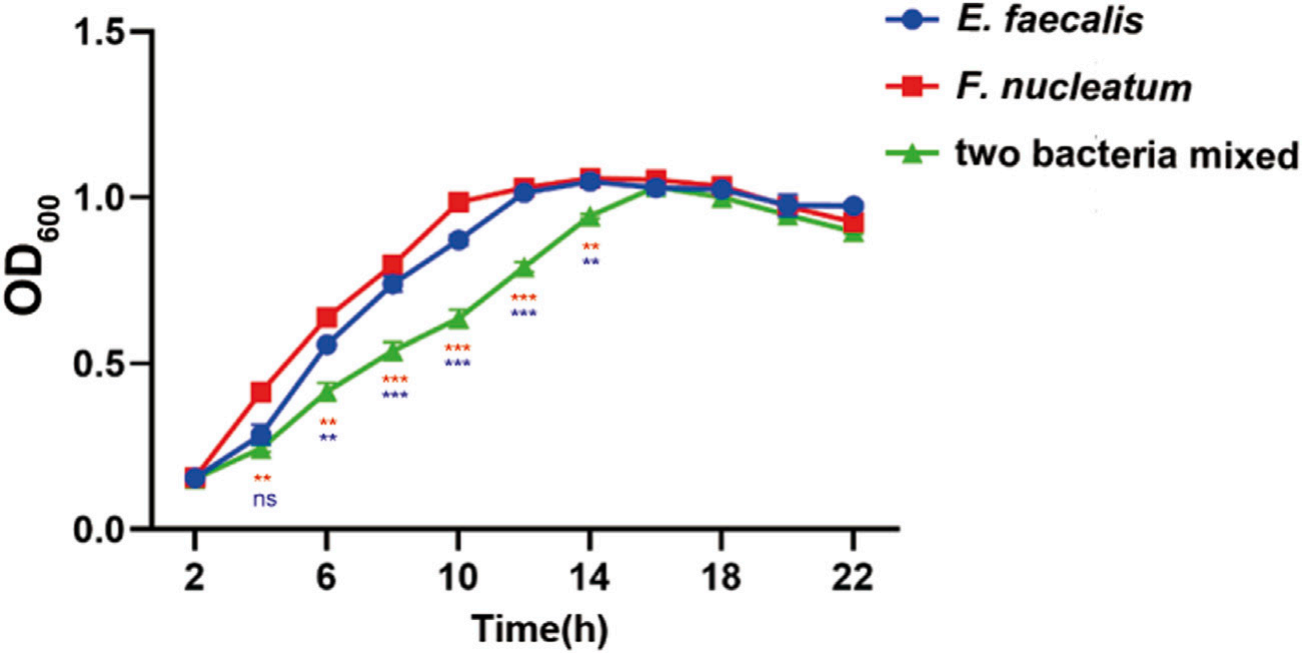
Growth curves of *E. faecalis* and *F. nucleatum* in single and mixed cultures. Data are presented as means ± standard deviations. Ns represent *p* > 0.05, *p < 0.05, **p < 0.01, ***p < 0.001. The red values represent the difference between the two species co-culture and the *F. nucleatum* cultured alone, the blue values represent the difference between the two species co-culture and the *E. faecalis* cultured alone.

The bacterial growth curve following a 24-hour culture revealed that *F. nucleatum* grew rapidly between 2 and 10 h, climbed steadily between 10 and 14 h, and stabilized after 14 h. *Enterococcus faecalis* showed rapid growth for 2–12 h before stabilizing. Over the course of 2–16 h, the OD _600_ of the mixed strains was lower than that of the single strains, with mixed bacterial growth tending to stabilize after approximately 16 h (Figure 2).

### 3.2 Bactericidal effect of antimicrobial peptides on preformed biofilms

**FIGURE 3:**
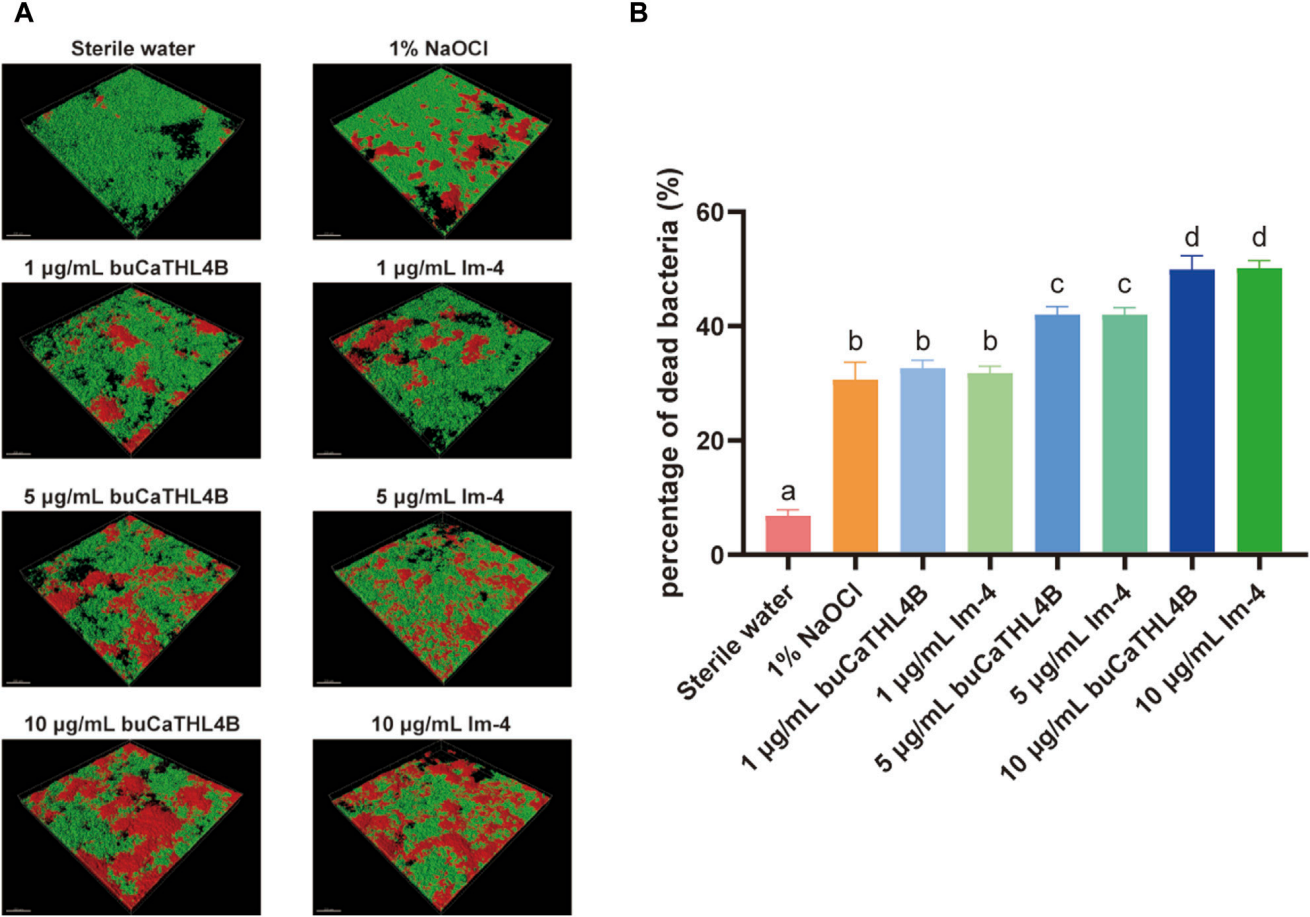
The bactericidal effect of buCaTHL4B and Im-4 on two-species biofilms. **(A)** Confocal microscopy images of two-species biofilms on HA discs treated with 10 μg/mL, 5 μg/mL, and 1 μg/mL buCaTHL4B or Im-4. The sterile water group served as blank control, and 1% NaOCl group as the positive control. **(B)** The proportion of dead bacteria as measured by viability staining and CLSM. Data are presented as means ± standard deviations. Different lowercase letters within each group indicate statistically significant difference (*p* < 0.05).

For the MIC, we observed that at a concentration as high as 80 μg/mL, neither peptide substantially inhibited the growth of *E. faecalis* and *F. nucleatum* (*p* > 0.05, Supplementary Figure S2). However, different concentrations of buCaTHL4B or Im-4 demonstrated obvious bactericidal effects on the 7-day biofilms (Figure 3A). The bactericidal rates corresponding to 10 μg/mL, 5 μg/mL, and 1 μg/mL concentrations of AMPs were 49.94% ± 2.39%, 42.03% ± 1.37%, and 32.66% ± 1.41% for the buCaTHL4B groups, and 50.18% ± 1.31%, 42.02% ± 1.22%, and 31.81% ± 1.21% for the Im-4 groups, respectively, in comparison to the sterile water controls (*p* < 0.05, Figure 3B). At a concentration of 1 μg/mL, the bactericidal rate of both AMPs was not significantly different from that of the 1% NaOCl group (*p* > 0.05, Figure 3B).

### 3.3 Antimicrobial peptides inhibit biofilm formation by CLSM

**FIGURE 4:**
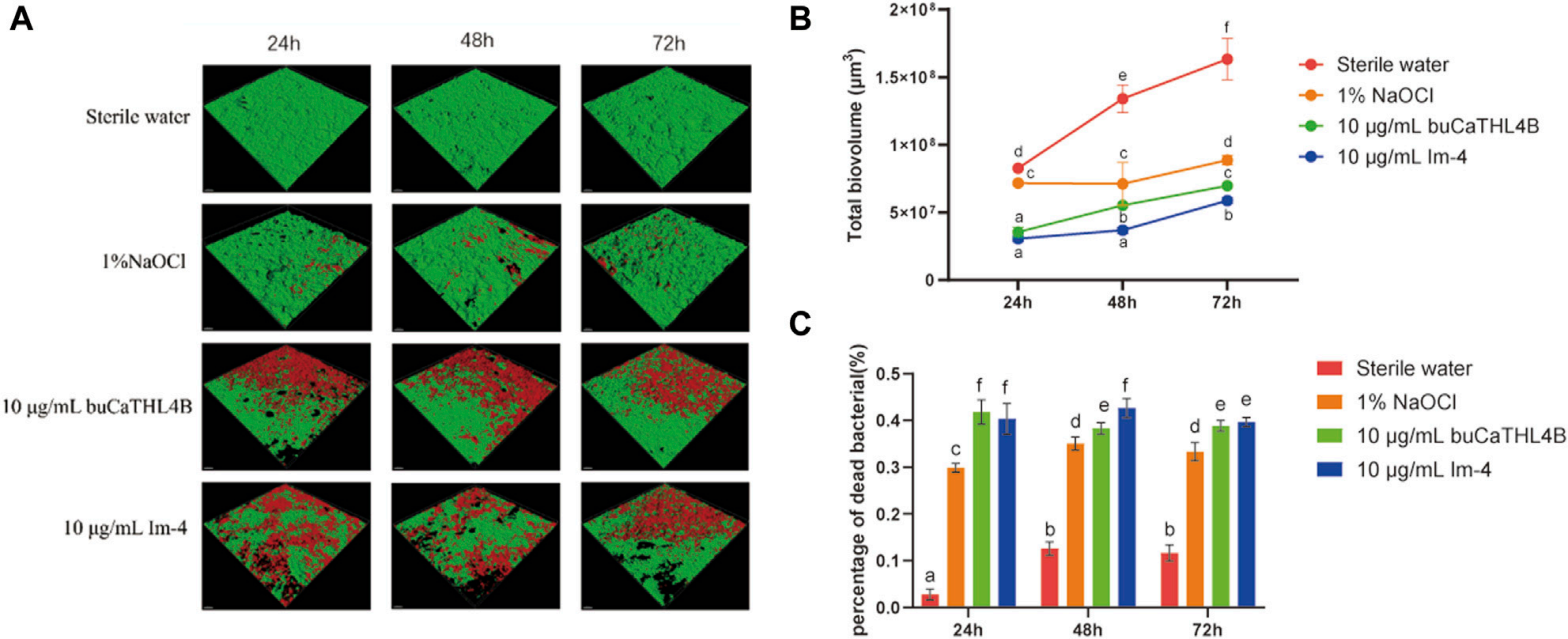
The antibiofilm effect of buCaTHL4B and Im-4 during the development of two-species biofilms. **(A)** Confocal microscopy images of biofilm development over 3 days in the presence of 10 μg/mL buCaTHL4B and Im-4. The sterile water group served as blank control, and 1% NaOCl group as the positive control. **(B)** The total biovolume of the biofilm formed over 3 days in the presence of 10 μg/mL buCaTHL4B and Im-4. Data are presented as means ± standard deviations. **(C)** The proportion of dead bacteria in the biofilm formed over 3 days in the presence of 10 μg/mL buCaTHL4B and Im-4. Data are presented as means ± standard deviations. Different lowercase letters within each group indicate statistically significant difference (*p* < 0.05).

The two-species biofilm formation was inhibited by the two peptides in inhibition experiments (Figure 4A). After 72 h of treatment, the biovolume of the two-species biofilm was significantly reduced by 10 μg/mL buCaTHL4B or Im-4, resulting in approximately 42.78% ± 3.55%, 40.34% ± 2.53%, and 44.74% ± 2.37% residual biofilm biovolume for buCaTHL4B groups, and 36.88% ± 2.10%, 26.87% ± 0.40%, and 37.52% ± 1.79% for Im-4 groups after 24, 48 and 72-hour time intervals, respectively, in comparison to the sterile water controls (*p* < 0.05, Figure 4B). Im-4 exhibited a stronger suppression effect than buCaTHL4B over the 72-hour period, with this difference being statistically significant (*p* < 0.05, Figure 4B).

The percentage of dead bacteria in the developed biofilms was estimated. Following 24, 48, and 72-hour time intervals, the bactericide rates for the buCaTHL4B groups were 41.80% ± 4.02%, 38.31% ± 1.91%, and 38.83% ± 1.78%, while for Im-4 groups were 40.32% ± 2.66%, 42.59% ± 3.24%, and 39.64% ± 1.59%, respectively. These rates were significantly higher than those of the sterile water group (*p* < 0.05). Only at 48-hour mark did the bactericidal effect of Im-4 surpass that of buCaTHL4B (*p* < 0.05), with no significant difference observed at the other time points (Figure 4C). The outcomes demonstrated that both Im-4 and buCaTHL4B could effectively prevent the formation of two-species biofilms.

### 3.4 Antimicrobial peptides inhibit biofilm formation by qPCR

**FIGURE 5:**
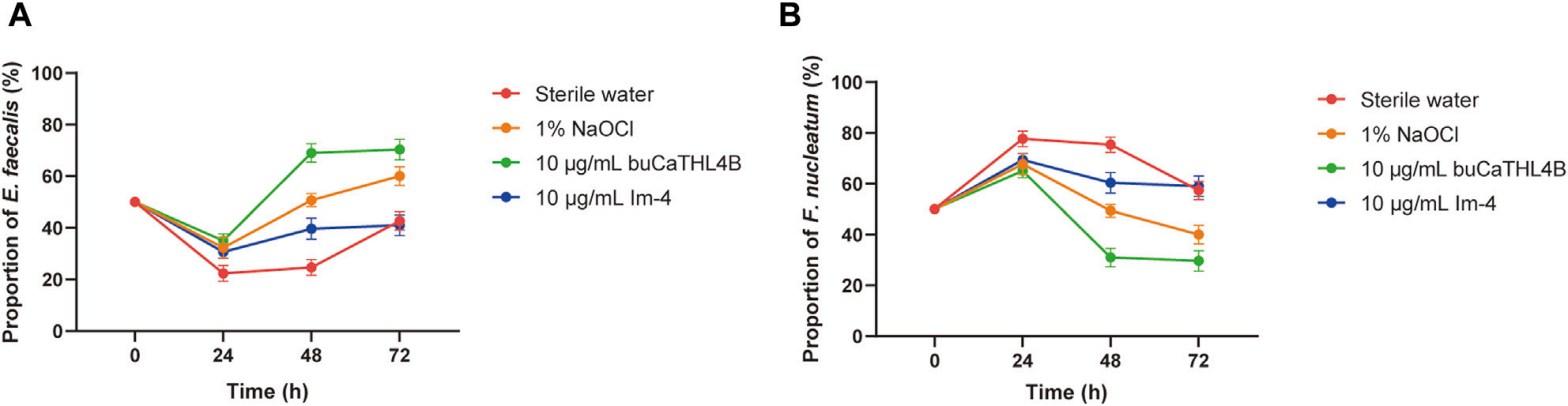
The proportion of *E. faecalis* **(A)** and *F. nucleatum* **(B)** in the biofilm of the different treatment groups at 24, 48, and 72 h exanimated by qPCR. Data are presented as means ± standard deviations.

The proportion of the two bacteria in the mixed biofilm following treatment with various agents was determined using a bacterial quantization algorithm. The percentage of *F. nucleatum* increased significantly in all groups at the 24-hour intervals. After 24 h of biofilm development, the proportion of bacterial species changed significantly. In the sterile water group, the proportion of *F. nucleatum* decreased, while the proportion of *E. faecalis* increased. In the buCaTHL4B group, *F. nucleatum* was found in lower proportions compared to *E. faecalis*, whereas in the Im-4 group, *F. nucleatum* was found in higher proportions. The bacterial proportion in both AMP-treated groups gradually stabilized after 48 h of treatment (Figure 5).

## 4 Discussion

This study evaluated the antibiofilm effects of buCaTHL4B and Im-4 on biofilms formed by *E. faecalis* and *F. nucleatum*. The results demonstrated that both buCaTHL4B and Im-4 at concentrations of 10 μg/mL exhibited significantly higher bactericidal activity compared to 1% NaOCl. Among the two peptides, Im-4 showed superior efficacy against biofilm formation over buCaTHL4B at the same concentration. Therefore, buCaTHL4B and Im-4 at 10 μg/mL are more effective against *E. faecalis* and *F. nucleatum* biofilms than the conventional irrigant solution NaOCl, the null hypothesis was rejected.

The formation of biofilms in a laboratory setting serves as a crucial model for studying microbial behavior and testing the efficacy of various disinfection techniques (Swimberghe et al., 2019). Pathogenic bacteria such as *F. nucleatum* and *E. faecalis* are commonly found in the root canal wall and dentin tubules as biofilms, contributing significantly to dental root canal infections, posing significant challenges for effective disinfection and treatment. (Razghonova et al., 2022; Hu Z. et al., 2023; Sheng et al., 2023). A two-species biofilm model was developed, mimicking the characteristics of an infected root canal biofilm. In mixed cultures, the time required for the bacteria to reach a stable state was longer compared to single cultures, consistent with previous studies. This delay suggests antagonistic interactions between the two strains, likely due to competition for limited nutrients within the medium. Both species compete for essential nutrients in the limited medium, leading to growth inhibition. This competition is more pronounced in a co-cultured environment than in single cultures (Chavez de Paz et al., 2015).

*Fusobacterium nucleatum* is known for having more adhesion proteins on its cell membrane compared with other bacteria. These proteins facilitate bacterial aggregation in the early stages of biofilm development (Lima et al., 2019). This early dominance was confirmed by qRT-PCR results, which showed an increase in the proportion of *F. nucleatum* in the biofilm during the initial 24-hour culture stage. This indicates that *F. nucleatum* occupies a dominant niche early in biofilm formation, potentially inhibiting *E. faecalis* growth. After 48 h of culturing, the proportion of *F. nucleatum* began to decrease. This shift can be explained by the creation of an acidic biofilm environment by *E. faecalis*, which inhibits the growth of *F. nucleatum* (Xiang et al., 2023). The sequencing of clinical samples supports these observations, showing *E. faecalis* prevalence in secondary infected root canals and *F. nucleatum* dominance in primary infections (Tennert et al., 2014; Bouillaguet et al., 2018; Qian et al., 2019). These findings highlight the importance of considering bacterial interactions when developing treatment strategies for root canal infections.

In the study, CLSM detection confirmed that *F. nucleatum* and *E. faecalis* could form a biofilm together after 7 days of co-culturing. This indicated that *F. nucleatum* can provide specific links or connections for other co-aggregative microorganisms during biofilm formation (Johnson et al., 2006). *E. faecalis* could co-adhere with *F. nucleatum*, facilitating biofilm formation, promoting interspecies communication, and enhancing bacterial survival in challenging environments (Yap et al., 2014). Further research revealed that *E. faecalis* physically binds to *F. nucleatum* in both planktonic and biofilm environments via the adhesion protein Fap2 (Xiang et al., 2023). Factors such as interactions between microorganisms significantly influence the composition of the microbiota. Laboratory biofilm models are indispensable for the preliminary assessment of root canal disinfection techniques. They provide a controlled environment to study biofilm dynamics, microbial interactions, and the efficacy of new treatments. Our findings highlight the importance of using such models to develop and refine strategies for managing biofilm-related infections in clinical dentistry.

In the study, the efficacy of buCaTHL4B and Im-4 was evaluated using various experimental methods to determine their bactericidal and inhibitory effects on mixed biofilms of *E. faecalis* and *F. nucleatum*. At concentrations significantly lower than 80 μg/mL (10 μg/mL), buCaTHL4B and Im-4 exhibited a significantly higher bactericidal rate in biofilms compared to the 1% NaOCl and markedly reduced biofilms volume. We employed the broth microdilution method to determine the MIC of buCaTHL4B and Im-4 against *E. faecalis* and *F. nucleatum*. However, buCaTHL4B and Im-4 may possess unique bactericidal mechanisms, such as rapidly killing bacteria by disrupting the cell membrane. This rapid and intense action might prevent the traditional MIC determination method from effectively detecting their activity (Wang et al., 2015). Among the two peptides, buCaTHL4B is a tryptophan-rich peptide (Brahma et al., 2015; D'Souza et al., 2021; Necelis et al., 2021). Tryptophan possesses potent hydrophobic qualities that can facilitate the amalgamation of peptides and lipid membranes, as well as cause bacterial mortality by disruption or passage through the bilayer (Shagaghi et al., 2016; Wang et al., 2021; Straus, 2024). Im-4 has been shown to work against Gram-positive bacteria, but the exact mechanism of action remains unclear (Guilhelmelli et al., 2016; Miyashita et al., 2017). Additionally, antimicrobial peptides may exhibit a concentration-dependent bactericidal effect, both buCaTHL4B and Im-4 demonstrated significant bactericidal effects at concentrations of 5 μg/mL and 1 μg/mL, though the efficacy decreased with lower concentrations.

In the 24, 48, and 72-hour experiments, the bactericidal rates in the 10 μg/mL buCaTHL4B and Im-4 treatment groups were significantly higher than those in the 1% NaOCl group. Although the bactericidal effects of the two antimicrobial peptides were similar at most time points, Im-4 exhibited a significantly higher bactericidal rate than buCaTHL4B at 48 h, indicating that Im-4 has a stronger biofilm inhibition capacity during certain time periods. This significant biofilm inhibition effect could be attributed to the unique mechanism of antimicrobial peptides, which cause cell death by disrupting bacterial cell membranes. The higher efficacy of Im-4 might be related to its stronger membrane-penetrating ability and may slow the development of pulp disease by preventing the biofilm from turning into secondary endodontic infections, as suggested by the reduced proportion of *E. faecalis* in the Im-4 group after 24 h. In addition, the inhibitory effect of buCaTHL4B and Im-4 on mixed biofilms of *E. faecalis* and *F. nucleatum* may also include interfering with bacterial signal transduction and hindering the formation of biofilm matrix. The specific role of these mechanisms needs to be further studied.

However, our study has some limitations. First, our model included only two highly abundant bacteria and was grown in a static environment, failing to capture the dynamic and intricate nature of the disease process inside infected root canals. Second, the *in vitro* data presented in this study may not fully replicate the *in vivo* situation. Therefore, further studies on isolated teeth with simulated root canal irrigation and clinical research are required to explore the actual efficacy of root canal irrigant with buCaTHL4B and Im-4 during root canal preparation. In addition, preclinical studies were conducted on animal models to verify the reliability and reproducibility of the laboratory results. This step is crucial to evaluate the effects of antimicrobial peptides in more complex biological settings. Third, more research, including molecular mechanism, is needed to fully understand the anti-biofilm processes of Im-4 and buCaTHL4B.

## 5 Conclusion

This study demonstrated the effective antibacterial and antibiofilm properties of both buCaTHL4B and Im-4, with Im-4 being more effective than buCaTHL4B in preventing biofilm formation. Im-4 regulates the amount of bacteria involved in biofilm production, which may slow the progression of pulp disease. Im-4 and buCaTHL4B are anticipated to be the potent components of a novel root canal irrigation solution.

## Data Availability

Publicly available datasets were analyzed in this study. This data can be found here: https://figshare.com/articles/code/LCMS_HPLC_buCaTHL4B_Im-4/26530180/2?file=48313975.
