# Supplementary material for: The effects of antimicrobial peptides buCaTHL4B and Im-4 on infectious root canal biofilms
Source: Front Bioeng Biotechnol. 2024 Aug 16;12:1409487. doi: 10.3389/fbioe.2024.1409487 (PMC11361941; doi:10.3389/fbioe.2024.1409487)
Supplement: Supplementary file 1 [file DataSheet1.PDF]

Supplementary Material

The Effects of Antimicrobial Peptides buCaTHL4B and Im-4 on Infectious Root Canal Biofilms

Ziqiu Hu[1,2,†], Haixia Ren[1,2,†], Yifan Min[3†], Yixin Li[1,2], Yuyuan Zhang[1,2], Min Mao[1,2], Weidong Leng[1,2*], Lingyun Xia[1,2*]

[1]Department of Stomatology, Taihe Hospital, Hubei University of Medicine, Shiyan, China

[2]Institute of Oral Diseases, School of Dentistry, Hubei University of Medicine, Shiyan, China

[3]Department of Stomatology, Zhushan County People's Hospital, Shiyan, China

*** Correspondence:**
Lingyun Xia; Weidong Leng
xialingyun200810@163.com; lwd35@163.com

[†]Ziqiu Hu, Haixia Ren, and Yifan Min contributed equally to this work and designated as co-first authors.

1 Supplementary Figure

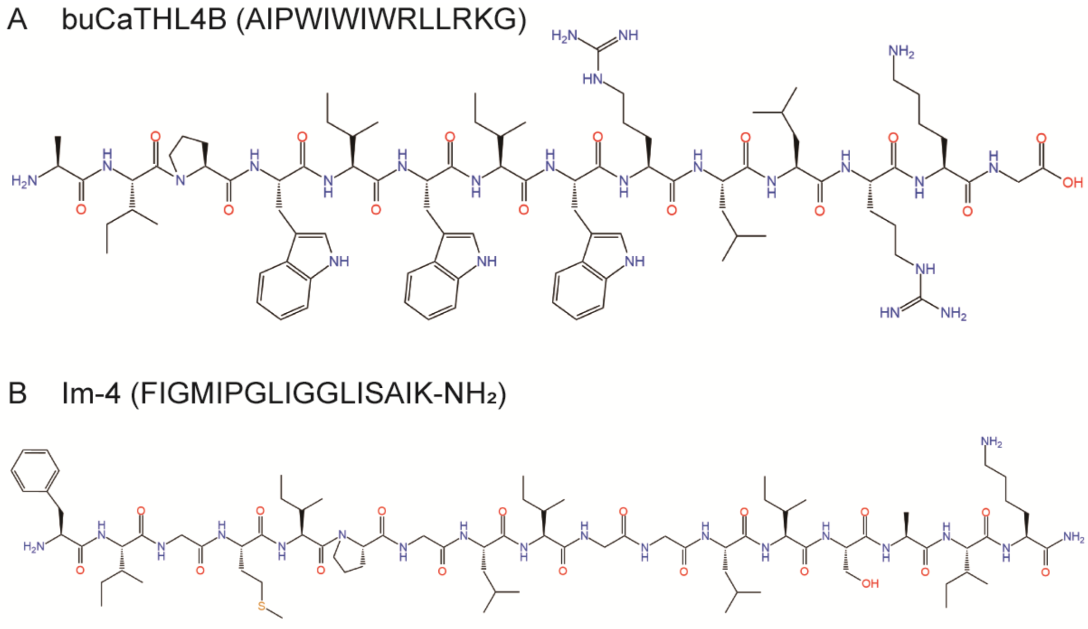

Supplementary Figure 1. Structures and sequences of buCaTHL4B (A) and Im-4 (B).

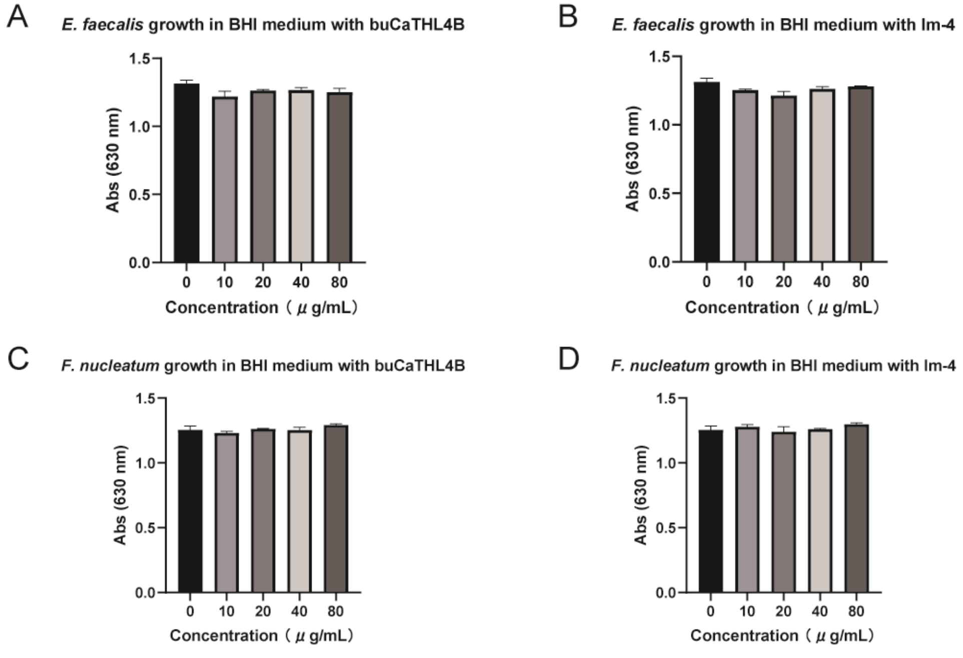

Supplementary Figure 2. *E. faecalis* and *F. nucleatum* were grown in BHI using 96-well polypropylene plates in the presence of increasing concentrations of buCaTHL4B and Im-4 (measured absorbance at 630 nm) was assessed after 24 hours.
